# Endoscopic management of an unusual clinical presentation of cholecystocolonic fistula

**DOI:** 10.1055/a-2873-7610

**Published:** 2026-06-29

**Authors:** Isabel Salvador, Sònia Fernández-Herrera, Mercè Güell, Alexander Osorio-Ramos, Carme Loras

**Affiliations:** 1 Endoscopy Unit 58955 Hospital Universitari MútuaTerrassaTerrassaSpain; 2 Surgery Unit 126691 Althaia Xarxa Assistencial Universitaria de ManresaManresaSpain; 3 Instituto de Salud Carlos III 468625 CIBEREHDMadridSpain

Cholecystoenteric fistulas (CCFs) are rare complications of gallstones; CCFs are the second most common type. CCFs may cause vague gastrointestinal symptoms or sepsis and diagnosis is difficult due to nonspecific findings 1 2 .

Gallstone-related colonic obstruction is an uncommon large bowel obstruction caused by a stone passing into the colon, usually through a CCF, most often affecting the sigmoid colon. Treatment is typically surgical, although endoscopic techniques are increasingly used 3 .

Both conditions mainly affect elderly patients and carry high morbidity and mortality due to delayed diagnosis and complex management 1 3 .

**Fig. 1:**
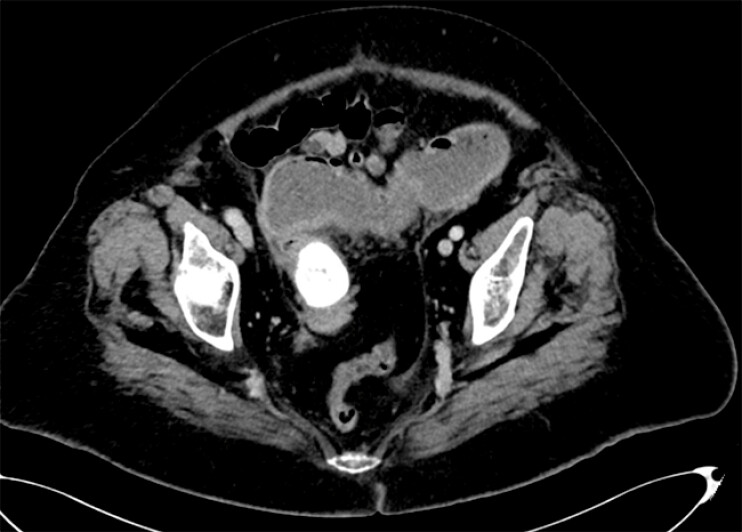
Abdominal CT revealing sigmoid obstruction caused by a 40-mm calcified intraluminal mass. CT, computed tomography.

We report the case of an 82-year-old woman presenting with constipation and vomiting. Laboratory tests showed leukocytosis and elevated C-reactive protein levels. Abdominal computed tomography (CT) revealed sigmoid obstruction caused by a 40-mm calcified intraluminal mass ( Fig. 1 ). Endoscopic decompression was attempted but failed due to a fixed sigmoid segment, and surgical resection with terminal colostomy was performed.

Postoperatively, the patient presented rising inflammatory markers and cholestatic enzymes. Repeat CT suggested acute cholecystitis and pneumobilia near the hepatic flexure. Magnetic resonance cholangiopancreatography confirmed cholelithiasis and a CCF.

**Fig. 2:**
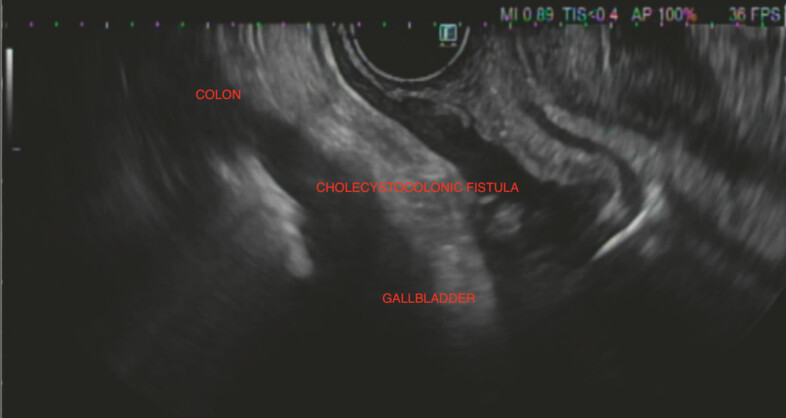
EUS demonstrating gallbladder with intraluminal air and communication with the colon. EUS, endoscopic ultrasound.

**Fig. 3:**
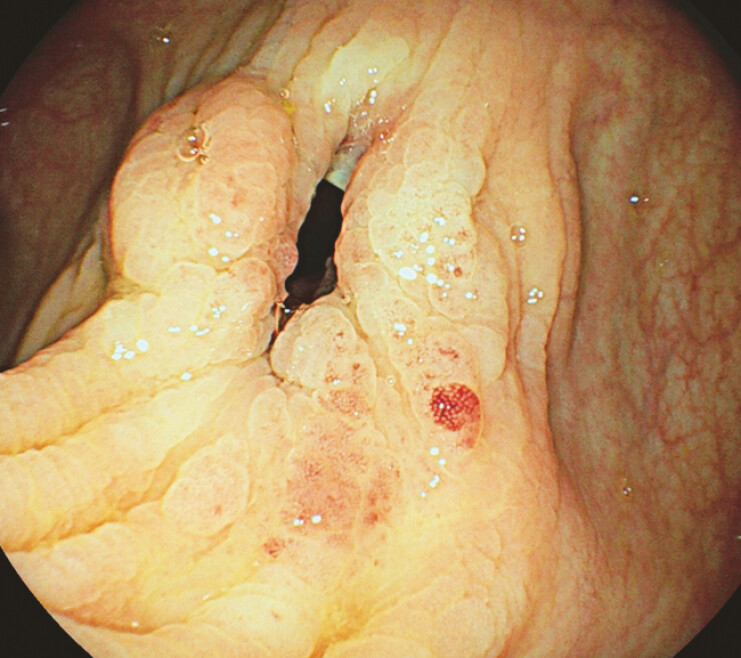
Lower gastrointestinal endoscopy revealing a fistulous orifice at the hepatic flexure.

**Fig. 4:**
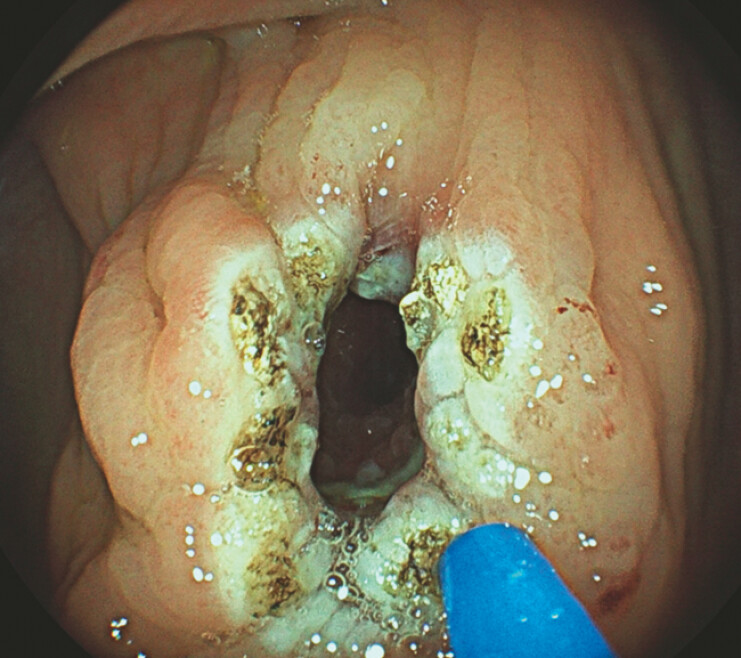
The fistula margins were treated with argon plasma coagulation.

**Fig. 5:**
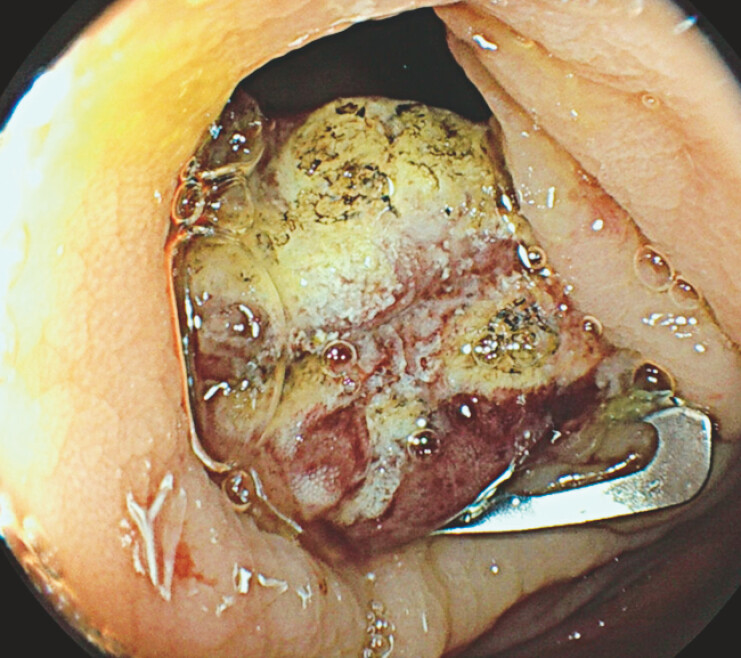
Fistula closure with a 14/6T over-the-scope clip.

Given her frailty, a minimally invasive endoscopic strategy was adopted. First, endoscopic ultrasound demonstrated a thick-walled gallbladder with intraluminal air and communication with the colon ( Fig. 2 ). Second, endoscopic retrograde cholangiopancreatography enabled biliary drainage and confirmed contrast passage from the gallbladder into the colon. Third, colonoscopy through the colostomy identified a 15-mm fistulous orifice at the hepatic flexure ( Fig. 3 ). After lavage, the fistula margins were treated with argon plasma coagulation ( Fig. 4 ), and closure was achieved using a 14/6T over-the-scope clip ( Fig. 5 ). The patient improved progressively and was discharged 6 days later.

This case illustrates the effectiveness of a combined multimodal endoscopic approach in diagnosing and achieving the complete resolution of a CCF ( Video 1 ). In frail patients who are poor surgical candidates, endoscopic management may constitute a safe and feasible therapeutic alternative, even in complex clinical scenarios.

Video case presentation and endoscopic management of the cholestocolonic fistula.Video 1

Endoscopy_UCTN_Code_CCL_1AD_2AG
